# Erythrabyssin ll is identified as a late-stage autophagy inhibitor reversing chemoresistance and promoting apoptosis in ovarian cancer

**DOI:** 10.1016/j.isci.2025.112801

**Published:** 2025-06-11

**Authors:** Jing Mo, Qiling Cai, Shanshan Chen, Jinlan Luo, Zhibiao Hu, Lili Su, Lulu Cheng, Lijie Huang, Shijia Liu, Xiangru Wang, Qinying Liu, Li Chen, Shuichun Mao, Yang Sun

**Affiliations:** 1 Department of Gynecology, Clinical Oncology School of Fujian Medical University, Fujian Cancer Hospital, Fuzhou 350014, China; 2 Fujian Provincial Key Laboratory of Tumor Biotherapy, Clinical Oncology School of Fujian Medical University, Fujian Cancer Hospital, Fuzhou 350014, China; 3 Fujian Provincial Key Laboratory of Medical Instrument and Pharmaceutical Technology, College of Biological Science and Technology, Fuzhou University, Fuzhou 350108, China; 4 School of Pharmacy, Jiangxi Medical College, Nanchang University, Nanchang 330006, China; 5 Obstetrics and Gynecology Department, Fujian Provincial Hospital, No. 134 East Street, Fuzhou 350001, China; 6 Interdisciplinary Institute for Medical Engineering, Fuzhou University, Fuzhou 350108, China

**Keywords:** Clinical pharmacy, Natural product chemistry, Biological sciences, Cancer

## Abstract

A growing body of research suggests that inhibition of autophagy may be a novel means of treating cancer and suppressing drug resistance. Therefore, a series of drugs derived from the *Erythrina crista-galli* Linn were screened in this study. Among them, the pterocarpan erythrabyssin II (EL-19) is a potent late-stage autophagy inhibitor, which could effectively block the fusion of autophagosome and lysosome, leading to the accumulation of autophagic substrates in both ovarian cancer A2780 and A2780/DDP cells. EL-19 did not impair the lysosomal pH and lysosomal enzyme activity. In addition, cell studies, and organoid experiments showed that EL-19 inhibited the value addition of A2780 and A2780/DDP cells, suppressed ovarian cancer organoid activity and induced apoptosis, and blocked cisplatin-induced protective autophagy in A2780/DDP cells. Combination therapy with DDP superior anti-tumor outcomes compared to monotherapies in animal models. In summary, EL-19 may be developed as an anticancer agent by blocking chemotherapy-induced protective autophagy.

## Highlights

• EL-19 inhibits chemotherapy-induced protective autophagy
• EL-19 blocks the fusion of autophagosome and lysosome
• EL-19 does not impair lysosomal pH or lysosomal enzyme activity
• The combination of EL-19 and cisplatin (DDP) outperforms DDP monotherapy in cancer treatment

## Graphical abstract

**Figure:**
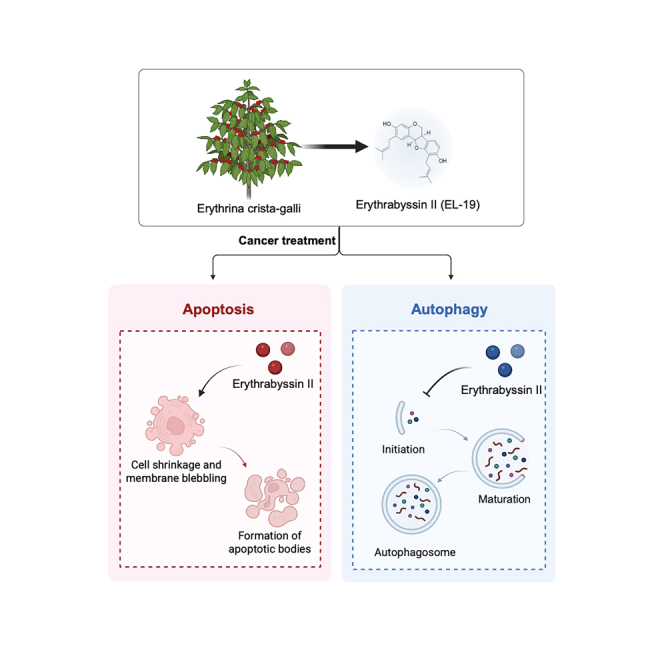

## Introduction

Ovarian cancer (OC) is a common gynecologic malignancy. Despite recent advances in diagnostic and treatment techniques, the 5-year survival rate for patients is still very low, the prognosis is far from ideal and the mortality still ranking first among gynecological tumors.^1^^,^^2^^,^^3^ The current first-line treatment for OC is mainly tumor-reducing surgery combined with postoperative platinum-based chemotherapy, but most patients will relapse due to platinum resistance and experience complications, such as fatigue, nausea, and vomiting, which seriously affect patients’ lives.^4^^,^^5^ Therefore, in order to improve the survival rate of OC patients and reduce the pain of patients’ treatment, it is necessary to develop low-toxicity and high-efficiency anti-tumor drugs and determine the best treatment strategy.

Macro-autophagy, abbreviated as autophagy, can transport dysfunctional or useless cell contents to lysosomes through the formation of autophagosomes with double-layered membranes so that they can be degraded by various hydrolytic enzymes in lysosomes.^6^^,^^7^ Autophagy is involved in a variety of physiological processes and plays a complex and diverse role in eukaryotic organisms, including cell growth, differentiation, and development.^8^ Abnormalities in cellular autophagy can disrupt the internal stability of cells, which can lead to the development of a variety of diseases, such as metabolic diseases, cancer, and diabetes.^9^^,^^10^ Several studies in recent years have shown that autophagy plays a dual role as an inducer of tumorigenesis or a tumor suppressor, and this paradoxical role depends on the different stages of cancer development.^11^ Generally, autophagy can prevent tumorigenesis by removing harmful substances and maintaining genomic stability.^12^ However, when tumors form, autophagy becomes a survival and protective mechanism for tumor cells, which can promote the development of cancer cell resistance during chemotherapy, thus resisting chemotherapeutic drug-mediated apoptosis and acting as a tumor pro-survival factor.^13^^,^^14^

Currently, the role of autophagy in tumors has led to widespread interest and research in autophagy inhibitors, and commonly used autophagy inhibitors include 3-methyladenine (3-MA), bafilomycin A1 (BafA1), LY294002, chloroquine (CQ) and its analogue hydroxychloroquine (HCQ).^15^ In addition, multiple autophagy inhibitors that directly target the autophagy formation phase have been identified, such as MRT68921.^16^ SB02024,^17^ Tioconazole,^18^ Lys05,^19^ Unfortunately, despite the current discovery of multiple autophagy inhibitors and numerous preclinical *in vivo* and *in vitro* experimental studies have been conducted, the only clinically available autophagy inhibitors are still CQ and HCQ, and some studies have shown that even CQ has limited ability to act *in vivo* due to retinal toxicity.^20^^,^^21^ Therefore, it is of great importance to develop autophagy inhibitors that are more specific, safer, with sufficient efficacy and clinically available.

Natural products have a long history of use as antitumor agents due to their properties. Natural products are usually of low toxicity, readily available and inexpensive, providing a valuable resource for the development of antitumor drugs.^22^^,^^23^ *Erythrina crista-galli* Linn, which grows mainly in tropical or subtropical regions, has attracted much attention because of its rich content of flavonoids and alkaloid compounds, for example, folitenol, erythrabyssin II and erycristagallin, and orientanol B and C have been isolated from it, and a number of substances have been found to have antimicrobial properties.^24^^,^^25^^,^^26^ In addition, it has been reported that the erythrabyssin II have concentration-dependent antiangiogenic effects on HRECs cells *in vitro*.^27^ However, few studies have investigated the role of compounds extracted from this plant on autophagy, so we sought to assess whether compounds isolated from it also have a regulatory effect on autophagy.

An organoid is an *in vitro* 3D culture model derived from stem cells or cancer cells that reproduces the biology of healthy or cancerous tissue.^28^ It takes less time, is easier to set up, and is less expensive than animal models. As early as 2009, organoid technology was introduced as a groundbreaking 3D primary tissue culture model and rapidly developed into a complex and promising preclinical model for cancer research.^29^ In recent years, organoids from OC and other solid tumors have been successfully established and applied to molecular biology research and drug screening experiments.^30^^,^^31^^,^^32^ So we sought to use organoids to participate in the evaluation of autophagy drugs.

In this study, by screening a series of compounds derived from the *Erythrina crista-galli* Linn, it was found that the pterocarpan erythrabyssin II (EL-19) could block autophagic flux without altering lysosomal pH and degradation function. As a novel late autophagy inhibitor, EL-19 could promote the apoptosis of OC organoid, OC A2780 cells and its cisplatin resistant type A2780/DDP cells, and the combination of EL-19 and DDP treatment showed synergistic anti-tumor effects on A2780/DDP cells both *in vivo* and *in vitro*. In summary, EL-19 could inhibit cisplatin induced autophagy and reverse cisplatin chemoresistance in cisplatin-resistant cells of OC, indicating its more significant anti-tumor effect and better tolerability *in vivo*.

## Results

### EL-19 is a novel autophagy regulator

**Figure 1:**
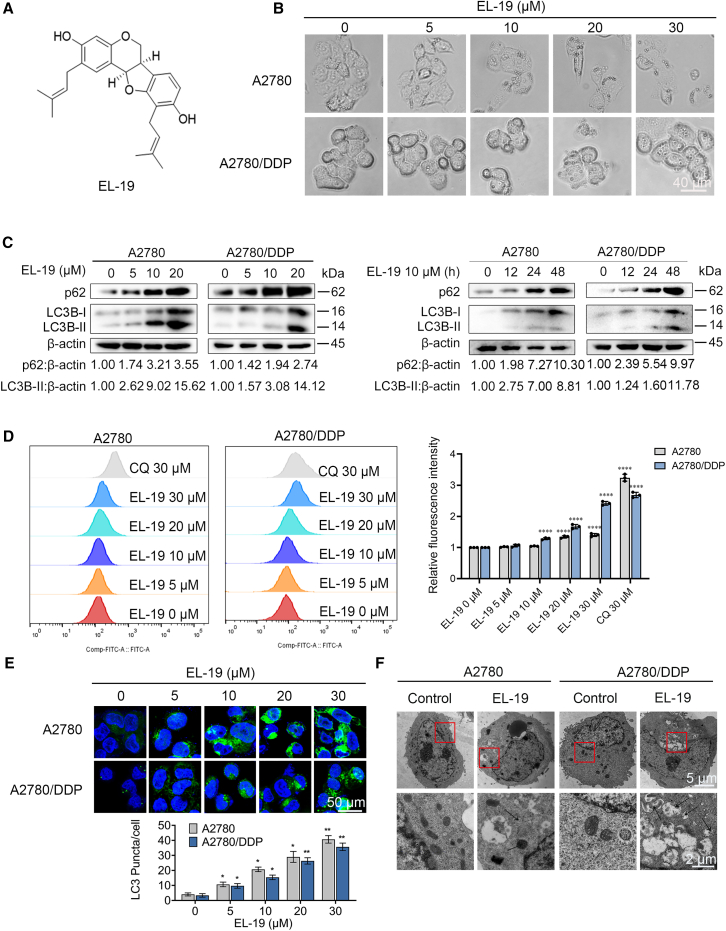
Identification of EL-19 regulates autophagy (A) The chemical structural formula of EL-19 with a molecular weight of 392 g/mol. (B) Light field images of A2780 and A2780/DDP cells treated with EL-19 (0–30 μM) for 24 h. Scale bar: 40 μm. (C) Western blot analysis of LC3B-ll and p62 proteins after treatment of A2780 and A2780/DDP cells with a specified concentration (0–20 μM) of EL-19 for 24 h or EL-19 (10 μM) for the indicated times (0–48 h). (D) After staining with CYTO-ID autophagy reagent, fluorescence intensity of A2780 and A2780/DDP cells treated with CQ (30 μM) or EL-19 (0–30 μM) for 24 h was analyzed using flow cytometry. Data are represented as mean ± SEM. *n* = 3, three biological replicates; ∗∗∗∗*p* < 0.0001 vs. EL-19 0 μM by two-way ANOVA. (E) Fluorescent photographs of GFP-LC3 spots in A2780 and A2780/DDP cells treated with EL-19 (0–30 μM) for 24 h. Scale bar: 50 μm. Cell nuclei were stained with DAPI. Histograms show quantification of the mean number of green spots per cell. Data are represented as mean ± SEM. *n* ≥ 10, three biological replicates; ∗*p* < 0.05, ∗∗*p* < 0.01 vs. EL-19 0 μM by two-way ANOVA. (F) Transmission electron microscopy pictures of A2780 and A2780/DDP cells treated with DMSO or EL-19 (20 μM) for 24 h. The second row are magnified images of the red boxes in the first row of images, and the image scale bars are 5 μm and 2 μm, respectively.

In order to discover new autophagy regulators, a series of compounds derived from *Erythrina crista-galli* Linn were screened. Among these compounds, EL-19 (Figures 1A and S1–S6) raised the cellular vacuolization both in OC A2780 cells and its drug-resistant type A2780/DDP cells with the increase of concentration gradient (Figure 1B), suggesting that EL-19 may regulate autophagy. To verify whether EL-19 could indeed regulate autophagy, we further examined the effect of the compound on the LC3-ll conversion. Protein immunoblotting results showed that EL-19 could elevate the LC3-ll protein level both in A2780 and A2780/DDP cells in a dose-dependent and time-dependent manner (Figure 1C). p62 can ubiquitinate protein aggregates and transfer them to the autophagy-lysosomal pathway for eventual degradation.^33^ Thus, elevated p62 protein level usually points to a blockade in autophagic flux. It was found that the expression level of p62 protein was elevated by EL-19 (Figure 1C), suggesting that EL-19 may inhibit autophagic flux rather than inducing autophagy.

Subsequently, we detected the regulatory function of EL-19 on autophagy by flow cytometry using the CYTO-ID autophagy kit. The results showed that EL-19 significantly increased the fluorescence intensity in A2780 and A2780/DDP cells, suggesting that El-19 may play a role in the regulation of autophagy, specifically, the effect of El-19 on autophagy of A2780/DDP cells was similar to that of CQ at 30 μM (Figure 1D). Next, the effects of EL-19 on autophagy in both A2780 and A2780/DDP cells were detected using confocal microscopy, and the results showed that the number of GFP-LC3 fluorescence dots in the cells was significantly enhanced after the action of EL-19, suggesting EL-19 might have a certain regulatory effect on autophagy (Figure 1E). Finally, we use transmission electron microscopy to observe the ultrastructural changes of cells after drug treatment, the results showed that compared with the control group, the cell vesicle structure of the EL-19 treated group was significantly increased and the formation of autophagosomes wrapped around the to-be-degraded material could be observed (Figure 1F). Overall, the previous results indicated that EL-19 could effectively regulate autophagy.

### EL-19 is a late-phase autophagy inhibitor but does not affect lysosomal pH or hydrolytic function

**Figure 2:**
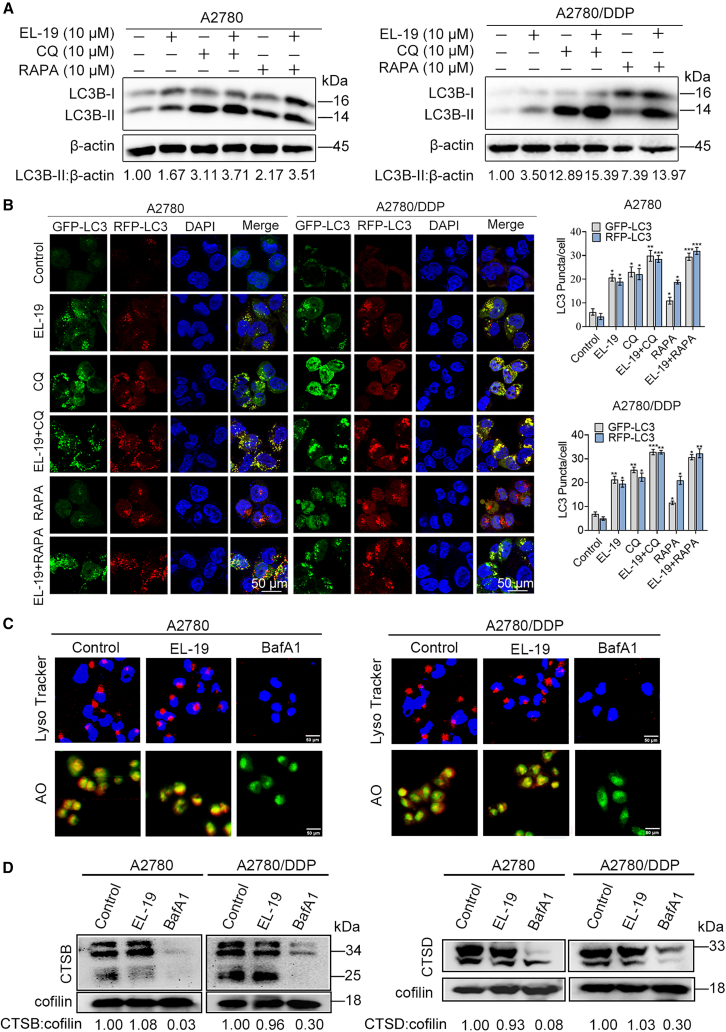
EL-19 blocks late autophagy but does not affect lysosomal function (A) A2780 and A2780/DDP cells were treated with 10 μM EL-19 and LC3B-ll protein level were analyzed by WB without or in the presence of 10 μM CQ or 10 μM RAPA for 24 h. (B) Laser confocal microscopy to observe fluorescence changes in A2780 and A2780/DDP cells transfected with mRFP-GFP-LC3 virus treated with EL-19 μM for 24 h without or in the presence of 10 μM CQ or 10 μM RAPA. The nuclei stained with DAPI. Scale bar: 50 μm. The average number of red and green dots per cell was counted. Data are represented as mean ± SEM. *n* ≥ 5, three biological replicates; ∗*p* < 0.05, ∗∗*p* < 0.01, ∗∗∗*p* < 0.001 vs. Control by two-way ANOVA. (C) A2780 and A2780/DDP cells were treated with 20 μM EL-19 or 100 nM BafA1 for 24 h, stained with LysoTracker red and acridine orange (AO), then fluorescence images were collected under inverted fluorescence microscope (Nikon Co., Ltd.). Nuclei were stained with DAPI. Scale bar: 50 μm. (D) WB analysis of CTSB and CTSD protein levels in A2780 or A2780/DDP cells treated with EL-19 (10 μM) or BafA1 (100 nM) for 24 h.

The elevated LC3-ll protein level and the increased number of GFP-LC3 fluorescent spots may either promote autophagosome formation or block autophagosome degradation.^34^ To verify which possibility it is, the effect of EL-19 on autophagic flux was further investigated. We analyzed the changes of LC3-ll protein level in A2780 and A2780/DDP cells treated with EL-19, the positive control rapamycin (RAPA) or the negative control chloroquine (CQ). The results showed that in the presence of CQ, EL-19 could not further significantly promote the LC3-ll protein level in both A2780 and A2780/DDP cells, whereas in the presence of RAPA, EL-19 could further promote the level of LC3-ll protein (Figure 2A), which suggests that EL-19 may inhibit autophagic lysosomal degradation. To further demonstrate the effect of EL-19 on autophagic flux, the role of EL-19 in autophagic flow was verified using the mRFP-GFP-LC3 dual-fluorescent viral assay. GFP-LC3 is a green fluorescent protein that is susceptible to fluorescence quenching in lysosomes, RFP-LC3 (red fluorescence) is not easily quenched, autophagic lysosomes emit only red fluorescence when autophagy is induced and more yellow fluorescent dots were observed when the process of autophagosome fusion with lysosomes was impaired or when lysosomal function was impaired. Our results showed that EL-19 significantly increased the number of yellow fluorescent spots in A2780 and A2780/DDP cells. In contrast, under autophagy induction (RAPA-treated group), more red fluorescent spots and very few green fluorescent spots were observed in the cells. Co-incubation of EL-19 with RAPA led to the further increase in yellow fluorescent spots compared with RAPA or EL-19 alone (Figure 2B), suggesting that EL-19 can block the autophagy-induced green fluorescence burst of GFP-LC3 to a certain extent. Therefore, the previous results indicated that EL-19 could block late autophagy.

Next, to determine whether EL-19 impairs the lysosomal acidic environment and thus disrupts degradation, we used LysoTracker red dye, which can be used for labeling and tracing of intracellular acidic organelles such as lysosomes. We used BafA1 as a positive control since it can disrupt autophagic flux by inhibiting lysosomal acidification. We observed that both control and EL-19-exposed group A2780 and A2780/DDP cells showed bright red fluorescence in the cytoplasm compared to the BafA1-treated group, which represented the accumulation of lysosomal acidity (Figure 2C), suggesting that EL-19 did not affect lysosomal PH. These results were further confirmed by AO staining (Figure 2C). In addition, lysosomes contain a large number of hydrolases that play a role in degradation. Therefore, the enzymatic activities of cathepsin D (CTSD) and cathepsin B (CTSB), which play an important role in degradation, were further examined. The results showed that the levels of CTSD protein and CTSB protein were significantly downregulated in BafA1-treated A2780 and A2780/DDP cells compared to the control group; however, the levels of CTSD protein and CTSB protein in the EL-19-treated group did not show significant changes (Figure 2D), suggesting that EL-19 did not inhibit the maturation of CTSD protein and CTSB protein, thereby blocking late autophagy.

### EL-19 suppresses autophagosome-lysosome fusion

**Figure 3:**
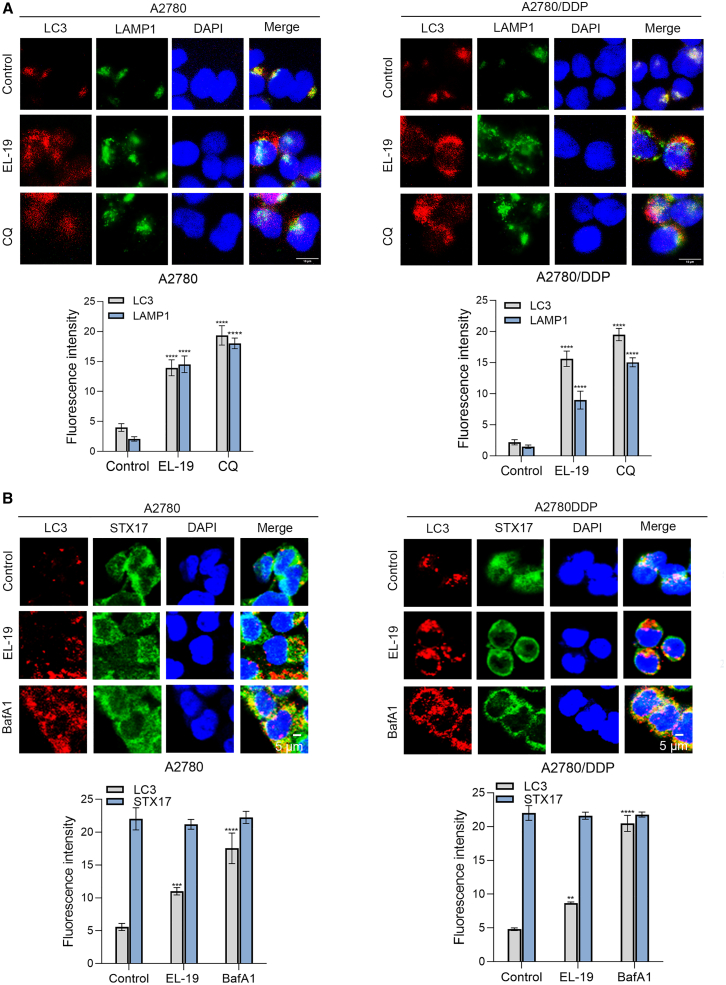
EL-19 suppresses autophagosome-lysosome fusion (A) Immunofluorescence photographs of the colocalization of LC3B (red) and LAMP1 (green) in A2780 cells and A2780/DDP cells treated with DMSO or EL-19 (20 μM) for 24 h. CQ (30 μM) treated cells were used as positive controls. Nuclei were stained with DAPI. Fluorescence images were collected under inverted fluorescence microscope. Scale bar: 10 μm. Data are represented as mean ± SEM. *n* = 3 (randomly selected three different areas), three biological replicates; ∗∗∗∗*p* < 0.0001 vs. Control by two-way ANOVA. (B) Immunofluorescence photographs of the colocalization of LC3B (red) and STX17 (green) in A2780 cells and A2780/DDP cells treated with EL-19 (20 μM) or BafA1 (100 nM) for 12 h. Nuclei were stained with DAPI. Fluorescence images were collected under confocal microscope. Scale bar: 5 μm. Data are represented as mean ± SEM. *n* = 3 (randomly selected three different areas), three biological replicates; ∗∗*p* < 0.01, ∗∗∗*p* < 0.001, ∗∗∗∗*p* < 0.0001 vs. Control by two-way ANOVA.

To confirm the effect of EL-19 on the fusion of autophagosomes with lysosomes, we used immunofluorescence to detect the co-localization of intracellular LC3B with LAMP1 (lysosome-associated membrane protein 1), a marker for lysosomal endosomal membranes. We could observe a clear separation of the red dots of LC3B and the green dots of LAMP1 in EL-19-treated A2780 and A2780/DDP cells, suggesting that the co-localization of LC3B with LAMP1 was reduced in EL-19-treated cells, similar to that in CQ-treated cells (Figure 3A). As shown in the quantitative fluorescence intensity statistics in Figure 3A, the level of LAMP1 was significantly increased in A2780 and A2780/DDP cells treated with EL-19 or CQ. Autophagic lysosome formation involves SNARE-mediated autophagosome-lysosome fusion, and STX17 is a key SNARE protein that mediates autophagosome maturation.^35^ Therefore, we further used immunofluorescence to detect the co-localization of LC3B with STX17 (Syntaxin 17) in cells. Our results were similar to previous reports in that after BafA1 for treatment, the red dots of LC3B and the green dots of STX17 significantly overlapped in A2780 and A2780/DDP cells, further increasing the co-localization compared to the control group, as this treatment prevented fusion by affecting lysosomal pH but did not affect autophagosomal SNARE assembly.^36^ Interestingly, although EL-19 treatment significantly reduced the co-localization of STX17 with LC3, the protein level of STX17 remained unaltered (Figure 3B). These results confirm that EL-19 inhibits intracellular fusion of autophagosomes with lysosomes.

### EL-19 inhibits the proliferation and induces apoptosis of A2780 and A2780/DDP cells

**Figure 4:**
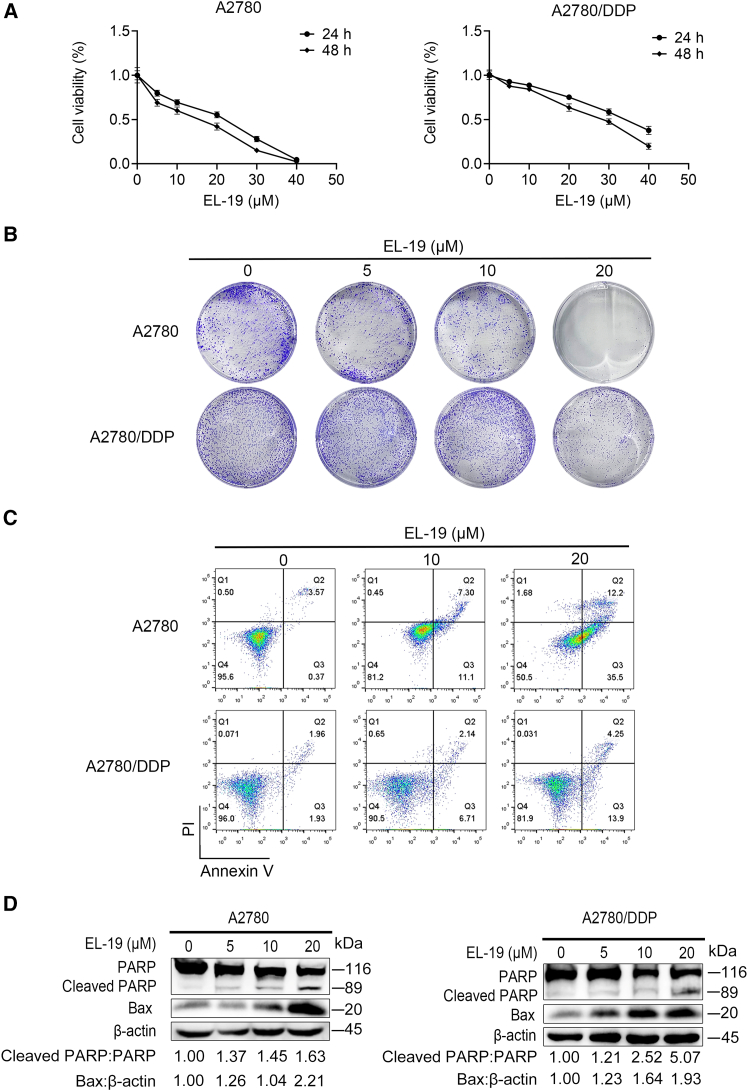
EL-19 inhibits proliferation and induces apoptosis in A2780 and A2780/DDP cell (A) CCK8 assay was used to determine the proliferation of A2780 and A2780/DDP cells treated with EL-19 (0–40 μM) for 24 or 48 h. (B) Colony formation of A2780 and A2780/DDP cells after 7 days treatment with EL-19 (0–20 μM). (C) The apoptosis of A2780 and A2780/DDP cells after EL-19 treatment with 0–20 μM for 24 h was analyzed by flow cytometry. (D) Western blot analysis of PARP, leaved PARP and Bax of A2780 and A2780/DDP cells treated with the indicated concentrations (0–20 μM) of EL-19 for 24 h.

Next, we investigated whether EL-19 has killing effect on OC cells *in vitro*, the effects of EL-19 on the viability of A2780 and A2780/DDP cells were evaluated by CCK8. As shown in Figure 4A, EL-19 could significantly inhibit the viability of A2780 and A2780/DDP cells in a dose-dependent and time-dependent manner. Consistent with this, colony formation assays showed that EL-19 significantly inhibited the proliferation of A2780 and A2780/DDP cells (Figure 4B). Then, we further examined whether EL-19 could promote apoptosis of OC cells by Annexin-V/PI method, found that EL-19 could promote apoptosis in A2780 and A2780/DDP cells in a concentration-dependent manner (Figure 4C). In order to further investigate the effect of EL-19 on apoptosis in OC cells, we detected the expressions of apoptosis-related proteins PARP and Bax by western blot. It was found that EL-19 inhibited PAPR protein and significantly enhanced the level of Bax protein in a dose-dependent manner, which further supported the pro-apoptotic effect of EL-19 (Figure 4D).

### EL-19 enhances the chemotherapy sensitivity of A2780/DDP cells to DDP by blocking autophagy

**Figure 5:**
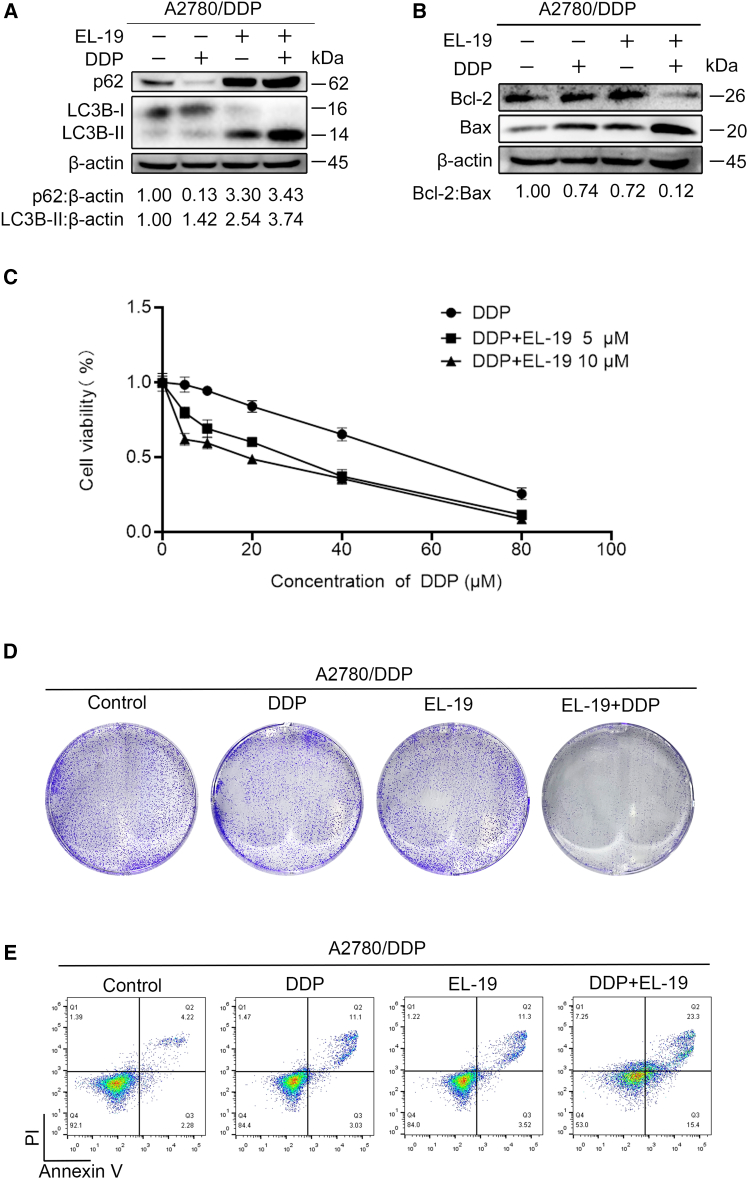
EL-19 enhance the anti-ovarian cancer activity *in vitro* by inhibiting DDP-induced autophagy (A) The changes of LC3B-ll and p62 proteins in A2780/DDP cells treated with 10 μM DDP for 48 h with or without 10 μM EL-19 were analyzed by western blot. (B) The changes of Bcl-2 and Bax proteins in A2780/DDP cells treated with 10 μM DDP for 48 h with or without 10 μM EL-19 were analyzed by western blot. (C) CCK8 assay was used to determine the proliferation of A2780/DDP cells treated with DDP (0–80 μM) for 48 h with or without EL-19 (5 μM and 10 μM). (D) Colony formation of A2780/DDP cells treatment with 10 μM DDP for 10 days with or without 10 μM EL-19. (E) The apoptosis of A2780/DDP cells treated with 10 μM DDP for 48 h with or without 10 μM EL-19 was analyzed by flow cytometry.

Studies have shown that tumor cells can resist drug-mediated apoptosis through autophagy, and inhibition of autophagy can promote apoptosis and inhibit proliferation.^37^ Cisplatin is a first-line agent for many solid tumors including OC; however, cisplatin resistance limits the therapeutic efficacy.^38^ Previous studies have demonstrated that autophagy may be one of the means of cancer cell escape from chemotherapy. Therefore, we are interested in whether the autophagy inhibitor EL-19 could restore the sensitivity of OC DDP-resistant cancer cells to DDP and enhance the cytotoxicity of DDP to OC DDP-resistant cancer cells. DDP treatment was found to increase the protein level of LC3-ll and decrease p62 protein level, indicating the onset of autophagy; whereas, EL-19 treatment increased the levels of both LC3-ll and p62 (Figure 5A), suggesting a blockade of autophagic degradation. We next investigated whether EL-19 could enhance chemosensitivity of OC cells by blocking DDP-induced autophagy. As shown in Figure 5B, DDP reduced Bcl-2 protein level and promoted Bax protein level in the presence of EL-19, suggesting that co-treatment of DDP with EL-19 promotes apoptosis in OC cells. Moreover, combination of DDP and EL-19 treatment reduced the viability of A2780/DDP cells more efficiently than DDP treatment alone (Figure 5C). The clonogenic assay results also showed that DDP restored its ability to inhibit A2780/DDP cells clonal formation in the presence of EL-19 (Figure 5D). Finally, Annexin V-PI experiments showed that co-treatment significantly induced A2780/DDP cell death compared to DDP or EL-19 treatment alone (Figure 5E). The previous data suggest that EL-19 could enhance its cytotoxicity to A2780/DDP through inhibiting DDP-induced autophagy *in vitro*.

### EL-19 inhibits the activity and induces apoptosis of ovarian cancer organoid

**Figure 6:**
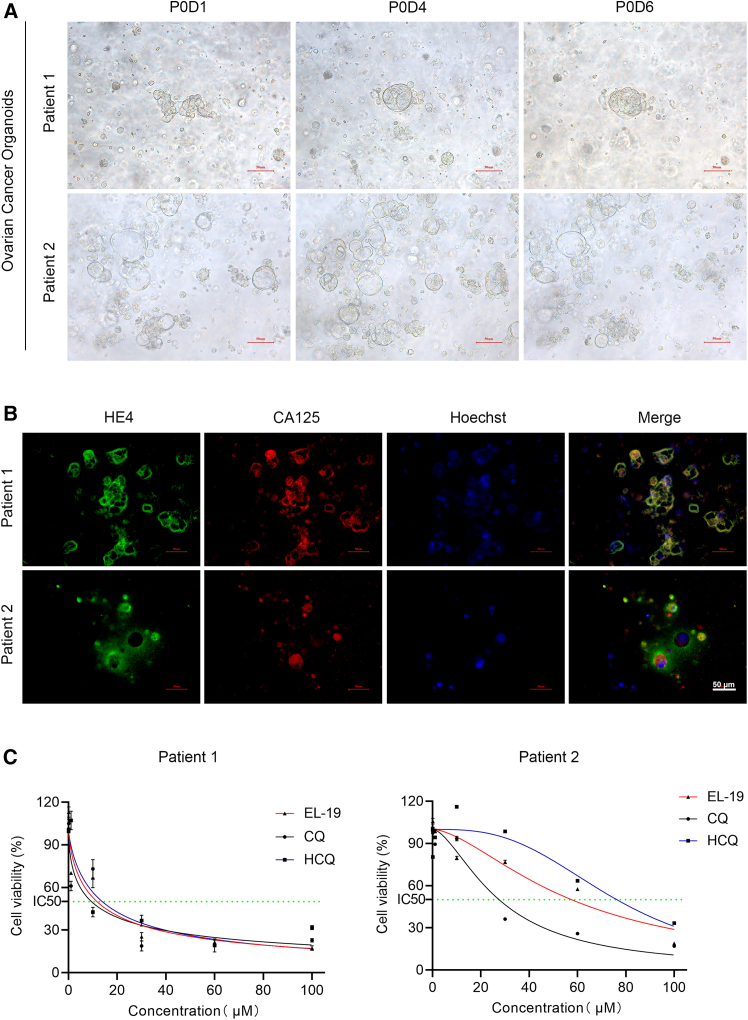
EL-19 inhibits activity and induces apoptosis of ovarian cancer organoid (A) Representative bright field microscopy images of patient-derived ovarian cancer organoids. OC organoids are derived from tumor tissue and ascites of patients (top to bottom). Scale bar: 50 μm. (B) Detection of the distribution of HE4 and CA125 proteins in OC organoids by immunofluorescence assay. Scale bar: 50 μm. (C) ATP assay was used to determine the activity of OC organoids treated with EL-19, CQ, HCQ (0.1–100 μM) for 48 h. The Sy.x derived from tumor tissue were 11.35 (EL-19), 14.49 (CQ), and 12.37 (HCQ), respectively. The Sy.x values of organoids derived from ascites were 8.479 (EL-19), 7.577 (CQ), and 10.72 (HCQ).

We selected two OC organoids of different origin for further verification. The patient 1 sample was derived from OC tumor tissue and patient 2 was derived from ascites. Within one week of culture, the size and morphology of OC organoids changed, and they showed different morphologies, such as vacuole and solid spherical (Figure 6A). We performed multiplex immunofluorescence staining of OC organoids, and staining with MUC16 and HE4 confirmed the OC origin of patient 1 and patient 2 (Figure 6B). To evaluate the effect of EL-19 in OC organoids, we utilized the luminescent cell viability assay to evaluate the effects of different concentrations of EL-19, CQ, and HCQ on OC organoid viability. As shown in Figures 6C and 6D, EL-19, CQ, and HCQ inhibited the viability of OC organoids in a dose-dependent manner. The IC50 values of organoids derived from tumor tissue were 11.69 (EL-19), 9.458 (CQ), and 13.89 (HCQ), respectively. And The IC50 values of organoids derived from ascites were 57.57 (EL-19), 27.53 (CQ), and 76.30 (HCQ). Therefore, it is easy to see that the efficacy of EL-19 is comparable to that of CQ or HCQ in each outcome group. And the efficacy of CQ is always superior to HCQ. Next, we performed apoptosis fluorescence staining on drug-treated OC organoids and found that EL-19, CQ, and HCQ all promoted apoptosis (Figure S7). The results also indicated that the organoids from different patients and different origins might react different from the same drugs, particularly the ascites were usually from advanced ovarian patients and showed more drug-resistant than the tumor tissue from the first treatment patients.

### EL-19 enhances the therapeutic effect of DDP by inhibiting autophagy in the A2780/DDP xenograft nude mouse model

**Figure 7:**
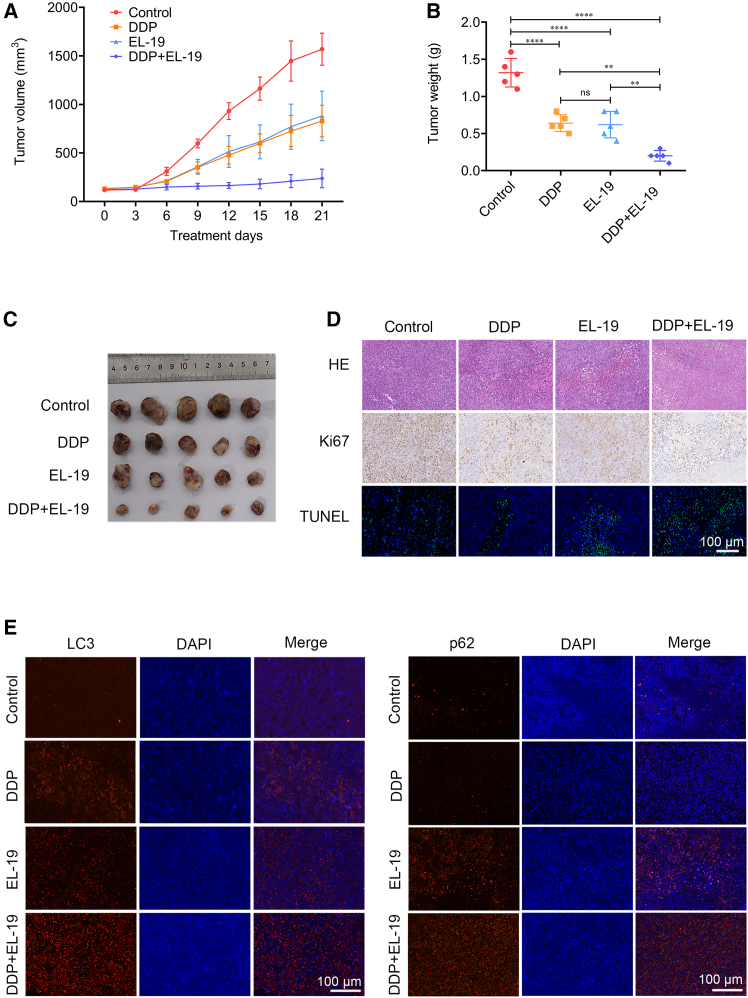
EL-19 enhances the therapeutic effect of DDP by inhibiting autophagy in the A2780/DDP xenograft nude mouse model (A) The tumor size was measured with a caliper every 3 days and the volume was calculated according to the formula (length × width^2^)/2. (B) The tumors were removed and weighed on the day the mice were killed. (C) Tumor images from the four groups on the day mice were sacrificed. (D) H&E and IHC staining were used to analyze the expression levels of Ki67 and TUNEL staining (green) in tumor sections. Scale bar: 100 μm. (E) Immunofluorescence staining representation of LC3B and p62 in tumors. Scale bar: 100 μm.

We have shown that EL-19 has an antitumor effect *in vitro*. Therefore, to assess whether EL-19 exhibits the same efficacy *in vivo*, an A2780/DDP nude mouse graft tumor model was constructed and the doses of EL-19 and DDP in this study were determined. After tumor graft formation, mice were intraperitoneally injected with saline, DDP (5 mg/kg), EL-19 (5 mg/kg), or DDP (5 mg/kg) + EL-19 (5 mg/kg) every 3 days for 3 weeks. As shown in Figures 7A–7C, the tumor size and weight increased significantly in the control group, but the tumor growth of the other three treatment groups was subjected to different degrees of inhibition. Specially, the combination treatment exhibited the slowest tumor growth and the best efficacy.

**Figure 8:**
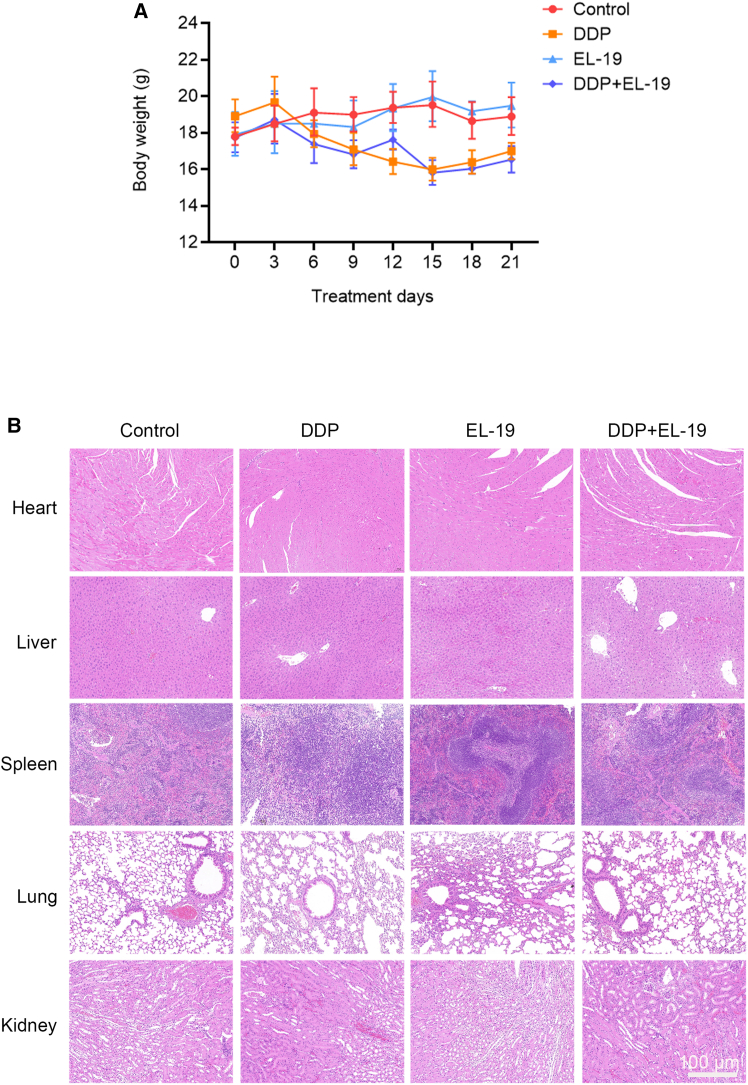
The safety examination of EL-19 *in vivo* (A) Body weight was measured every 3 days. (B) H&E staining of heart, liver, spleen, lung, and kidney. Scale bar: 100 μm.

As shown in Figure 7D, the IHC and immunofluorescent staining results showed the combination treatment of DDP and EL-19 further significantly inhibited Ki67 expression and increased TUNEL expression in tumor, indicating that the combination treatment efficiently promoted tumor apoptosis and inhibited tumor proliferation. Immunofluorescent results also showed that DDP decrease the expression of p62 and indeed induced autophagy in tumor, whereas, EL-19 significantly enhanced the expression of LC3-II and p62 and successfully inhibited DDP-induced autophagy in tumor tissues (Figure 7E). Throughout the experiment, EL-19 had no significant toxicity to mice, the average body weight of mice in the EL-19 group did not decrease, while the DDP group showed obvious weight loss (Figure 8A). The results of HE sections of major organs, including heart, liver, spleen, lungs, and kidneys of nude mice, showed that there was no significant damage to the major organs of mice after EL-19 drug treatment, however, mice treated with DDP showed kidney damage, and the group of DDP and EL-19 combined also showed kidney damage but the symptoms were not further enhanced (Figure 8B), which indicated that EL-19 has a certain degree of biosafety. In general, EL-19 could restore the tumor sensitivity to the chemotherapy drug DDP *in vivo* without safety risk.

## Discussion

Under external stimuli, such as hypoxia, lack of nutrients, and metabolic stress, autophagy can survive and maintain intracellular homeostasis through its ability to recycle cellular contents.^39^ Cancer cells can obtain the energy and nutrients necessary for survival by activating autophagy, which in turn promotes tumor development.^40^ In addition, autophagy is also an important cause of resistance to cancer chemotherapy, elevated levels of autophagy can promote the development of multidrug resistance in tumors, whereas inhibition of autophagy enhances therapeutic efficacy.^41^^,^^42^ In recent years, although more and more autophagy modulators have been discovered and studied, there are still few available clinically. Therefore, it is very important to develop autophagy modulators with higher specificity, safety, clinical applicability, and fuller efficacy.

**Figure 9:**
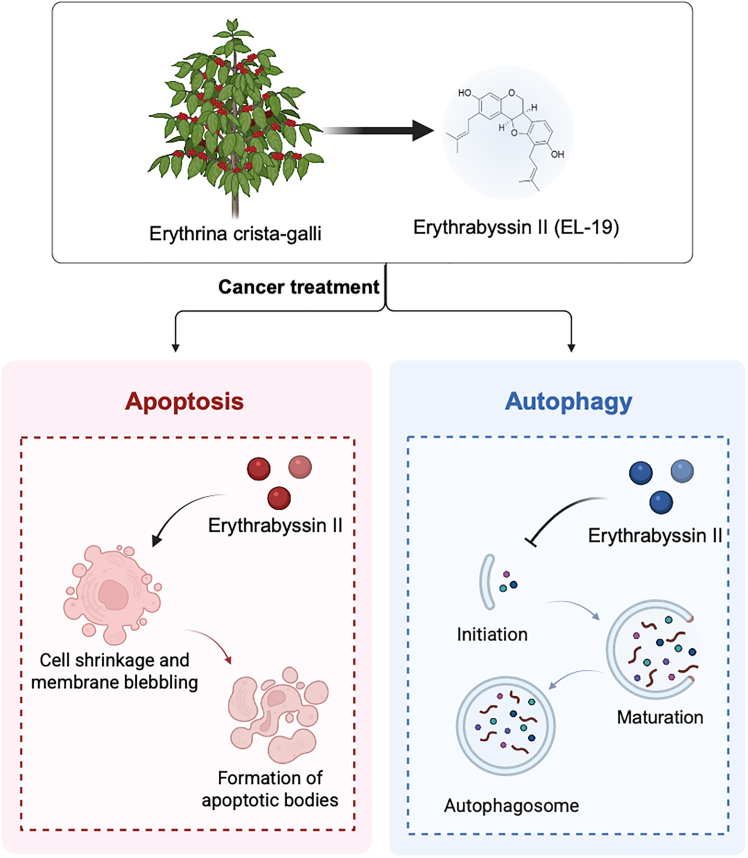
Mechanism of EL-19 regulating autophagy to reverse drug resistance of ovarian cancer EL-19 was extracted from cockscombs Erythrina, which could inhibit autophagy and promote apoptosis of ovarian cancer cells. In A2780/DDP xenografted nude mouse models, EL-19 reversed ovarian cancer resistance.

Natural products provide a rich source not only for antitumor drugs but also for the development of autophagy modulators.^43^ erythrabyssin II (EL-19) is isolated and extracted mainly from the roots of *Erythrina crista-galli* L, which has been shown in previous studies to have promising antiangiogenic effects. In our study, we confirm that EL-19 is a novel autophagy regulator and further demonstrated that EL-19 blocked late autophagy and inhibited substrate degradation. EL-19 exhibited antitumor effects and extremely strong autophagy inhibition in A2780 and A2780/DDP cells *in vitro* and in OC xenograft nude mouse model *in vivo* (Figure 9).

In preclinical studies, patient-derived organoids (PDOs) serve as potent surrogates for understanding diseases and exploring new treatment options. By faithfully encapsulating the complex cellular structure and function of primitive tissues, PDO provides an extraordinary platform for exploring disease mechanisms and they are playing an increasingly critical role in preclinical drug development.^44^ Thus, using OC organoids, we confirmed that EL-19 is a novel autophagy regulator. Then, we also compared EL-19, CQ, and HCQ in OC organoids, and confirmed that these three drugs have similar anti-tumor effects, and the inhibitory effect on cell viability increases with the increase of drug concentration within a certain drug concentration.

Chemoresistance induced by single-agent immunotherapy usually leads to treatment failure, affecting the lives and survival of most patients, recent studies have shown that combination therapy has shown great benefit in clinical care and is one of the keys to improving survival rates and improving prognosis.^45^^,^^46^ The classical chemotherapeutic drug DDP, used to treat OC, can lead to chemoresistance and poor patient prognosis.^47^ Therefore, after demonstrating that EL-19 is an autophagy inhibitor, we further investigated whether inhibition of autophagy could reverse resistance and improve efficacy of OC treatment. We have verified that EL-19 and DDP alone have anti-tumor effects, but the combination treatment of EL-19 and DDP demonstrated a significant coordinated effect that could go further to significantly induce OC cell death. EL-19 exhibited low toxicity and inhibited tumor growth *in vivo* when used alone, in addition, the results of DDP drug treatment showed that chemotherapeutic drug treatment induced protective autophagy in tumor tissues and apoptosis was increased in tumor tissues by the combination of EL-19.

Furthermore, we verified through a series of experiments that EL-19 blocked late autophagy. p62 appeared to be enriched as well as lysosomal acidic but hydrolytic proteases CTSD and CTSB did not change after EL-19 treatment, suggesting that EL-19 does not act as an autophagy inhibitor by impairing lysosomal function. Interestingly, we found that EL-19 could prevent the fusion of autophagosomes with lysosomes by reducing the intracellular co-localization of LC3B with LAMP1 and the co-localization of LC3B with STX17, which blocked the autophagic flow. The targets of EL-19 in autophagic flow still need to be further explored on the basis of this study.

In conclusion, we found that pterocarpan EL-19 is a novel late-stage autophagy inhibitor for OC both *in vivo* and *in vitro*.

### Limitations of the study

In this study, we demonstrate that EL-19 could inhibit autophagy and reverse cisplatin chemoresistance in cisplatin-resistant cells of OC, indicating that it possesses good anti-tumor effects. However, whether other EL-19 analog extracted from chickweed acanthopanax has the same autophagy regulating effect, and whether the autophagy regulating effect of different structural EL-19 analogs on cells is similar, still need further studies.

## Resource availability

### Lead contact

Further information and requests for resources and reagents should be directed to and will be fulfilled by the lead contact, Yang Sun (sunyangfjch@163.com).

### Materials availability

This study did not generate new unique reagents.

### Data and code availability

• Original data reported in this paper will be shared by the lead contact upon request.
• This paper does not report original code.
• Any additional information required to reanalyze the data reported in this paper is available from the lead contact upon request.

## Acknowledgments

10.13039/501100001809National Natural Science Foundation of China (grant nos: 82374081, 82060633, and 81860616); Joint Funds for the Innovation of Science and Technology, Fujian Province, China (grant no: 2021Y9209); Natural Science Foundation of Jiangxi Province (grant nos: 20212ACB206034 and 20202BAB206084). Natural Science Foundation of Fujian (grant no: 2021J01608 and 2024J011070); High-level Talents Training Project of Fujian Cancer Hospital (grant no: 2022YNG04); The Fuzhou University Testing Fund of Precious Apparatus (grant no: 2024T019).

## Author contributions

Conceptualization, J.M.; methodology, J.M.; investigation, J.M., Q.C., S.C., J.L., L.S., L.C., L.H., S.L., and X.W.; formal analysis, Q.C., S.C., J.L., L.H., S.L., and X.W.; writing—original draft, J.M., Q.C., Q.L., and L.C.; writing—review and editing, J.M., J.L., L.C., Q.L., L.S., and L.C.; funding acquisition, S.M. and Y.S.; resources, Z.H.; project administration, Q.L., L.C., S.M., and Y.S.; supervision, Y.S.

## Declaration of interests

The authors declare that they have no known competing financial interest or personal relationships that could have appeared to influence the work reported in this paper.

## STAR★Methods

### Key resources table

**Table:** 

REAGENT or RESOURCE	SOURCE	IDENTIFIER
**Antibodies**		

Anti-LC3B	CST	Cat#83506; RRID:AB_2800018
Anti- LAMP1	CST	Cat# 9091; RRID:AB_2687579
Anti- Cleaved PARP	CST	Cat# 9541; RRID:AB_331426
Anti- Bcl-2	CST	Cat# 3498; RRID:AB_1903907
Anti- Bax	CST	Cat# 2772; RRID:AB_10695870
Anti- PARP	CST	Cat# 9542; RRID:AB_2160739
Anti-β-actin	CST	Cat# 4967; RRID:AB_330288
Anti- p62	Abcam	Cat# ab207305; RRID:AB_2885112
Anti- CTSD	Boster	Cat# BM1577
Anti- CTSB	Boster	Cat# A01456-3; RRID:AB_3081445
Anti- Cofilin	Proteintech	Cat# 66057-1-Ig; RRID:AB_11043339
Anti- HE4	Proteintech	Cat# 66557-1-Ig; RRID:AB_2881918
Anti-MUC16	MCE	Cat# HY-P81044; RRID:AB_3103039
Coralite 594-conjugated goat anti-mouse IgG	Proteintech	Cat# SA00013-3; RRID:AB_2797133
Coralite 488-conjugated goat anti-rabbit IgG	Proteintech	Cat# SA00013-2; RRID:AB_2797132
Anti- STX17	Abclonal	Cat# A17174; RRID:AB_2772449

**Chemicals, peptides, and recombinant proteins**		

EL-19	Nanchang University	N/A
Cisplatin	Sigma	Cat# 232120
Chloroquine	Shanghai Aladdin	Cat# C129284
Rapamycin	MedChemExpress	Cat# HY-10219
Bafilomycin A1	MedChemExpress	Cat# HY-N6738
Puromycin	InvivoGen	Cat# ant-pr-1
Paraformaldehyde tissue fixative	Shanghai Yuanye	Cat# W12136
Hydroxychloroquine	MCE	Cat# HY-W031727
DAPI	Solarbio	Cat# C0065
Beyo3DTM Hoechst	Beyotime	Cat# C1345M
Matrigel	Corning	Cat#354230

**Critical commercial assays**		

Caspase 3/7 Activity Apoptosis Assay Kit	Beijing Baiao	Cat# KFS208
High-Grade Serous Ovarian Cancer Organoid Kit	bioGenous Biotech	Cat# K2167-HS
CellCounting-Lite 3D Luminescent Cell Viability Assay	Vazyme	Cat# DD1102-02
BCA Protein Assay Kit	Beyotime	Cat# P0012
Annexin V-FITC Apoptosis Detection Kit	Beyotime	Cat# C1062L
CYTO-ID® Autophagy detection kit	Enzo	Cat# ENZ-51031-K200
Cell Counting Kit-8	MCE	Cat# HY-K0301

**Experimental models: Cell lines**		

Human:A2780	KeyGEN Biotech	Cat#KGG3271-1
Human:A2780/DDP	KeyGEN Biotech	Cat# KGG3508-1

**Experimental models: Organisms**		

BALB/c Nude mice	GuangdongYaokang Biotechnology Co., Ltd	N/A

**Software and algorithms**		

GraphPad Prism software 8.0	GraphPad Software, Inc.	https://www.graphpad-prism.cn/
FlowJo software 10.5.3	Becton, Dickinson & Company	https://www.flowjo.com/
ImageJ software	National Institutes of Health	https://imagej.en.softonic.com/
Adobe Photoshop	Adobe Company	https://www.adobe.com/

### Experimental model and study participant details

#### Cell lines

A2780 and A2780/DDP cell lines were purchased from KeyGEN Biotech. All cell lines were maintained in RPMI 1640 (HyClone) supplemented with 1% penicillin (HyClone), 1% streptomycin (HyClone) and 10% fetal bovine serum (GEMINI) in a humidified incubator at 37°C under 5% CO_2_ atmosphere.

#### Mice

BALB/c female nude mice aged 4–6 weeks were purchased from Guangdong Yaokang Biotechnology Co., Ltd. The mice were used for tumor xenograft model, they were housed in clean, improved barrier animal facilities and fed a clean commercial mouse diet under controlled light/dark cycling and controlled temperature. The mouse study procedures *in vivo* were performed according to the Animal Care Committee of Fujian Provincial Hospital.

#### Tumor xenograft experiment

Each mouse in the tumor model was injected subcutaneously with 5 × 10^6^ A2780/DDP cells (100 μL). When the tumor volume reached about 100 mm^3^, the mice were randomly divided into four groups: control group, DDP group, EL-19 group and EL-19+DDP group, five mice in each group. The injection dose of DDP and EL-19 was 5mg/kg. All groups of mice were injected intraperitoneally and administered once every 3 days. Tumor length (L) and width (W) were measured by vernier calipers on 3 days, used the formula: L × (W)^2^/2. Mice were euthanized 21 days after treatment and tumors were weighed at the end of treatment.

### Method details

#### Bright-field microscopic photography

A2780 and A2780/DDP cells were incubated on 24-well glass-covered slides for a certain period of time and then treated with EL-19 (0, 5, 10, 20 or 30 μM) for 24 h, Photographs were obtained using inverted fluorescence microscope (Olympus Corporation).

#### Western blotting analysis

Total proteins of treated A2780 or A2780/DDP cells were extracted with modified RIPA buffer (Shanghai Epizyme Biomedical Technology Co., Ltd) containing 1 mM PMSF. Protein quantification was performed using BCA Protein Assay Kit (Beyotime Biotechnology). Protein bands were separated using SDS-PAGE gels (Shanghai Epizyme Biomedical Technology Co., Ltd), followed by transfer to nitrocellulose membranes. Membranes blocked with 5% w/v skimmed milk powder (Beyotime Biotechnology) in Tween-containing PBST blocking buffer at room temperature for 1 h and then were incubated overnight at 4°C with primary antibodies, washed, followed by incubation with HRP-conjugated goat anti-rabbit IgG or HRP-conjugated goat anti-mouse IgG secondary antibody at room temperature for 1 h. Finally, the bands were visualized using an ultrasensitive chemiluminescent solution in a FluorChem E digital darkroom system (ProteinSimple; Bio-Techne).

#### Viral infection

Spreaded A2780 cells or A2780/DDP cells in 6-well plates and wait until the cell density reaches 50%–60%, sucking out the old medium, add 1 mL of complete medium containing mRFP-GFP-LC3 dual fluorescent viruses and Polybrene (Genomeditech Co., Ltd.) according to the instructions provided by the company, and then make up 1 mL of complete medium in each well after 24 h, and then culture for another 48 h. Add puromycin to the screen until the rate of viral infection in the cells is more than 90%.

#### Autophagy flux assay

A2780 or A2780/DDP cells transfected with mRFP-GFP-LC3 dual fluorescent virus were cultured in confocal dishes and treated with 10 μM EL-19, 10 μM RAPA or 10 μM CQ in combination or alone for 24 h. Cells with 4% paraformaldehyde (Solarbio Science & Technology Co., Ltd.) fixed for 15 min and nuclei were stained with DAPI (Solarbio Science & Technology Co., Ltd.) staining solution for 10 min according to the instructions. Photographs were taken and recorded under the confocal microscope (Nikon Co., Ltd.).

#### CYTO-ID autophagy assay

A2780 and A2780/DDP cells were spread into 6-well plates and treated with 30 μM CQ or EL-19 (5, 10, 20, 30) for 24 h Collected cells and incubated with pre-configured CYTO-ID staining solution (Enzo) for 30 min at 37°C in the dark, washed twice with buffer and finally resuspended in buffer. Fluorescence was measured using a flow cytometer (BD Biosciences).

#### CCK8 assay

∼3,000 A2780 or A2780/DDP cells were spread into 96-well plates and treated with serial dilutions of DMSO or EL-19 (5, 10, 20, 30 and 40 μM) for 24 h or 48 h. In addition, ∼3,000 A2780/DDP cells were inoculated into 96-well plates and treated with DDP (5, 10, 20, 40 and 80 μM) in combination or alone with EL-19 (5, 10 μM) for 48 h. After treatment, CCK8 solution (supplied with the kit, MedChemExpress) was added to each well and the absorbance was analyzed at 450 nm using an enzyme marker (SH-1000; Corona Electric Co., Ltd.).

#### Colony formation assay

∼1.0 × 10^3^ A2780 or A2780/DDP cells were spread into 24-well plates After the cells were adherent, with complete medium containing the desired concentration of drug. The cells were cultured for 7–10 days according to the cell growth condition and when obvious colonies could be observed by naked eyes, the well plates were washed with PBS buffer and then were fixed with 4% paraformaldehyde for 15 min, followed by adding diluted crystal violet solution (Beyotime Biotechnology) according to the instructions of reagent vendors to stain the cells for about 5 min for photographs and recordings.

#### Apoptosis assays

A2780 or A2780/DDP cells were inoculated into 6-well plates at 2.5 × 10^5^ cells/well and treated with serial dilutions of EL-19 (5,10 and 20 μM) for 24 h. In addition, 2.5 × 10^5^ A2780/DDP cells were inoculated into 6-well plates and treated with EL-19 (10 μM), DDP (10 μM) alone or EL-19 (10 μM) in combination with DDP (10 μM) for 48 h. After the treatment, the cells were gently blown with buffer solution under light-avoidance conditions and successively added with 5 μL AnnexinV-FITC and 10 μL PI solution (Beyotime Biotechnology). Finally, analyzed for apoptosis using FACS can flow cytometer (BD Biosciences).

#### LysoTracker red staining

A2780 or A2780/DDP (∼8×10^4^) was spread in a confocal dish, treated with 20 μM EL-19 or 100 nM BafA1 for 24 h. The sample was incubated with LysoTracker red staining solution (Beyotime Biotechnology) at 37°C for 40 min and washed twice with PBS. 1 mL of medium and 5 μL of DAPI-stained nuclei solution were added, and the sample was incubated for 8–10 min. At the end of incubation, the staining solution was aspirated and gently washed twice with PBS. Laser confocal microscope was used to examine and take pictures (Nikon Co., Ltd.).

#### AO staining

A2780 or A2780/DDP (∼1×10^5^) was spread in 12-well glass-covered chamber slides and then treated with 20 μM EL-19 or 100 nM BafA1 for 24 h. Following treatment, the cells were incubated with AO for 15 min at 37°C in the dark. Fluorescent images were obtained using an inverted fluorescence microscope (Nikon Co., Ltd.).

#### Immunofluorescence assay

The A2780 or A2780/DDP (∼1×10^5^) was spread in 12-well plates and treated with 20 μM EL-19, 30 μM CQ or 100 nM BafA1 for 24 h or 12 h. Following treatment, the cells were fixed with 4% paraformaldehyde for 15 min, then Saponin (Beyotime Biotechnology) was added to treat the slides of cells for 10 min at room temperature. Next, blocked with fetal bovine serum (3%) and incubated with anti-LC3B (1:200), LAMP1 (1:300) and STX17 (1:200) primary antibodies at 4°C overnight. The slides of cells were then incubated with CoraLite 594-conjugated goat anti-mouse IgG (1:200) and CoraLite 488-conjugated goat anti-rabbit IgG (1:200) at room temperature for 1 h in the dark and nuclei were stained with DAPI (Beyotime Biotechnology). Laser confocal microscope (Nikon Co., Ltd.) or Olympus inverted fluorescence microscope (Olympus Corporation) was used to take pictures. Images were randomly captured.

#### Transmission electron microscopy (TEM)

EL-19 treated and untreated cells were collected and fixed in 2.5% glutaraldehyde (Phygene) at 4°C for more than 24 h. Then sent the samples to the Institute of Quality Standards and Testing Technology for Agro-Products, Fujian Academy of Agriculture Science for transmission electron microscopy imaging.

#### Biological tissue sections testing

After resection of the tumor and the organs, the tissues were fixed with 4% paraformaldehyde tissue fixative. Finally, the tissues were sent to Hangzhou Yanqu Information Technology Co., Ltd for the H&E staining, Ki67 immunohistochemical analysisand immunofluorescence analysis of TUNEL, LC3 and p62.

#### Ovarian cancer (OC) organoids culture

A patient-derived ovarian cancer tissue specimen and an ascites fluid specimen were digested and lysed to produce cell aggregates, mixed with Matrigel to maintain a 3D structure in a complete medium containing nutrients, hormones and required growth factors, and then cultured in an incubator at 37°C and 5% CO_2。_All studies involving human tissue were performed with approval from Fujian Cancer Ethics Committee with informed consent obtained from patients. The ethics review number is K2024-277-01.

#### Bright-field microscopic photography

The morphological changes in the growth of ovarian cancer organoids are recorded within a 24-well plate and photographed using an inverted fluorescence microscope. Ovarian cancer organoids were treated with EL-19, CQ, HCQ (0, 30, 100 μm)^33^ for 48 h in 96-well plates, stained with Capase3/7 live-cell detection reagent and Beyo3D Hoechst, respectively, and photographed using an inverted fluorescence microscope. Ovarian cancer organoids were incubated with MUC16 antibody and HE4 Monoclonal antibody overnight in 24-well plates, incubated with corresponding secondary antibodies for 1–2 h, and then stained with Beyo3D Hoechst, and photographed using an inverted fluorescence microscope.

#### ATP assay

Approximately 2000 organoid cells per well were seeded into 96-well plates, and after one week of culture, EL-19, CQ, and HCQ were diluted with medium at concentrations of 0, 0.1, 1, 10, 30, 60, and 100 μM and treated for 48 h. CellCounting-Lite 3D Luminescent Cell Viability Solution was added to each well and detected with a microplate reader.

#### Apoptosis assay

Approximately 2000 organoid cells per well were seeded into 96-well plates, cultured for one week, ovarian cancer organoids were treated with EL-19, CQ, HCQ (0, 30, 100 μM) for 48 h, Capase3/7 live cell detection reagent and Beyo3D Hoechst were added for staining, respectively, and photographed using inverted fluorescence microscopy.

#### Multiple immunofluorescence

Approximately 1×10^5^ organoid cells per well were seeded into 24-well plates, incubated overnight with MUC16 antibody and HE4 Monoclonal antibody after one week of culture, incubated for 1–2 h with the corresponding secondary antibody, and then stained with Beyo3DTM Hoechst, and photographed using an inverted fluorescence microscope.

#### Statistical analysis

Statistical analyses were performed using GraphPad Prism 8 software (GraphPad Software, Inc.) All results are expressed as mean (SEM) ± standard error. Statistical significance was assessed using two-way analysis of variance (ANOVA). *p* < 0.05 was considered to indicate a statistically significant difference.
