# Supplementary material for: Erythrabyssin ll is identified as a late-stage autophagy inhibitor reversing chemoresistance and promoting apoptosis in ovarian cancer
Source: iScience. 2025 Jun 11;28(7):112801. doi: 10.1016/j.isci.2025.112801 (PMC12269293; doi:10.1016/j.isci.2025.112801)
Supplement: Document S1. Figures S1–S7 [file mmc1.pdf]

iScience, Volume *28*

Supplemental information

Erythrabyssin II is identified as a late-stage autophagy inhibitor reversing chemoresistance and promoting apoptosis in ovarian cancer

Jing Mo, Qiling Cai, Shanshan Chen, Jinlan Luo, Zhibiao Hu, Lili Su, Lulu Cheng, Lijie Huang, Shijia Liu, Xiangru Wang, Qinying Liu, Li Chen, Shuichun Mao, and Yang Sun

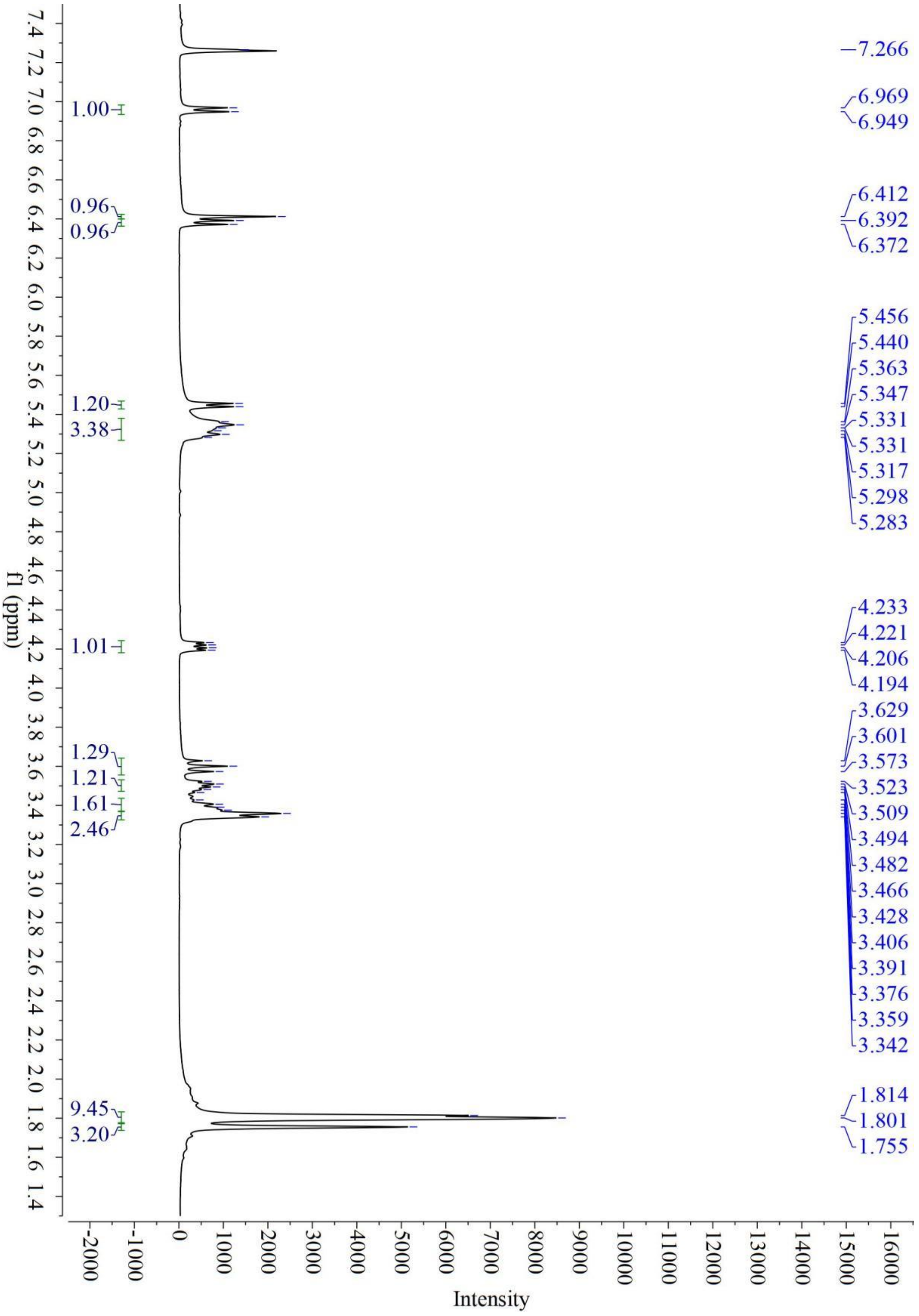

Figure S1. ^{1}H NMR ($CDCl_3$) spectrum of EL-19

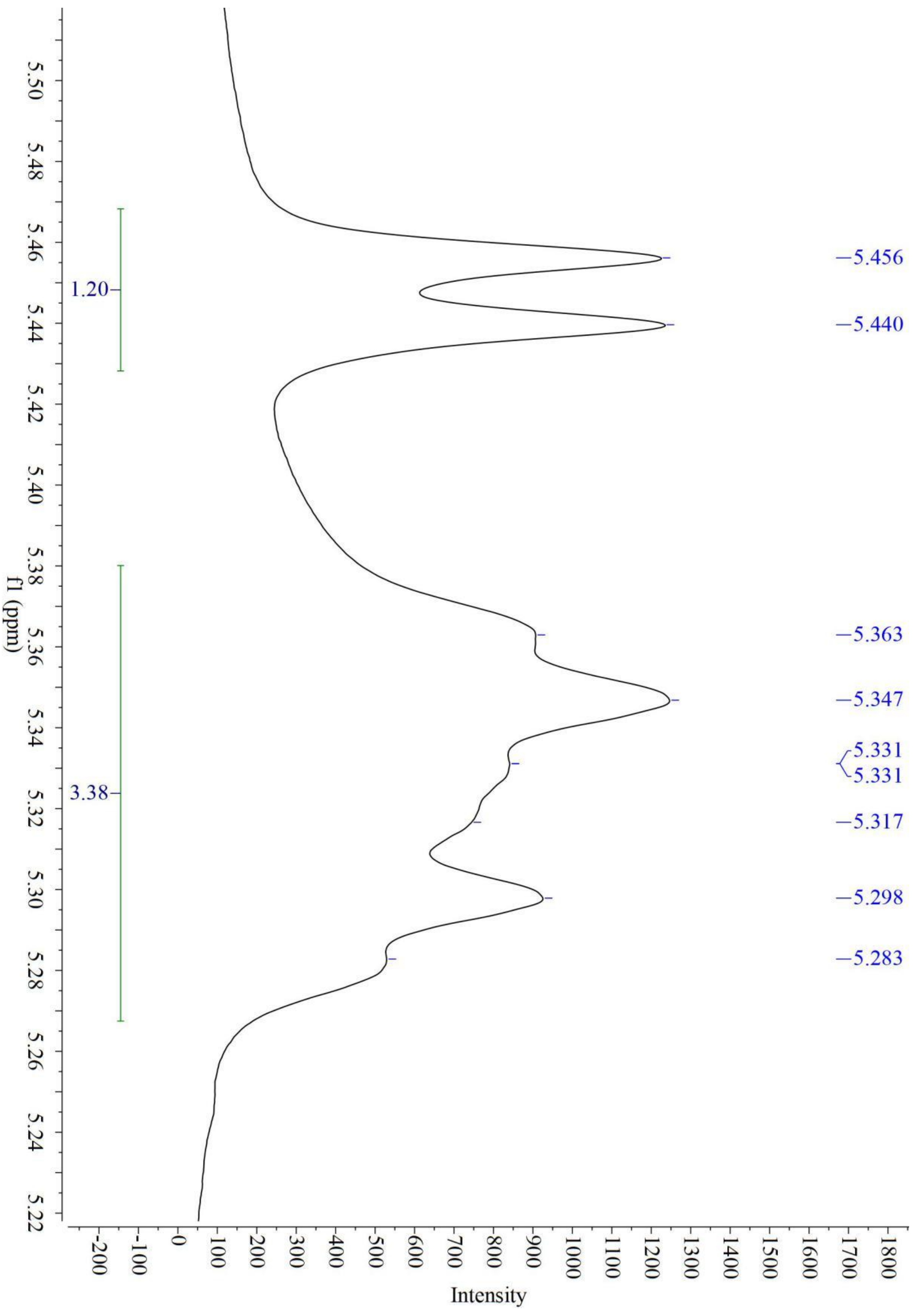

Figure S2. Magnified ^{1}H NMR ($CDCl_3$) spectrum of EL-19

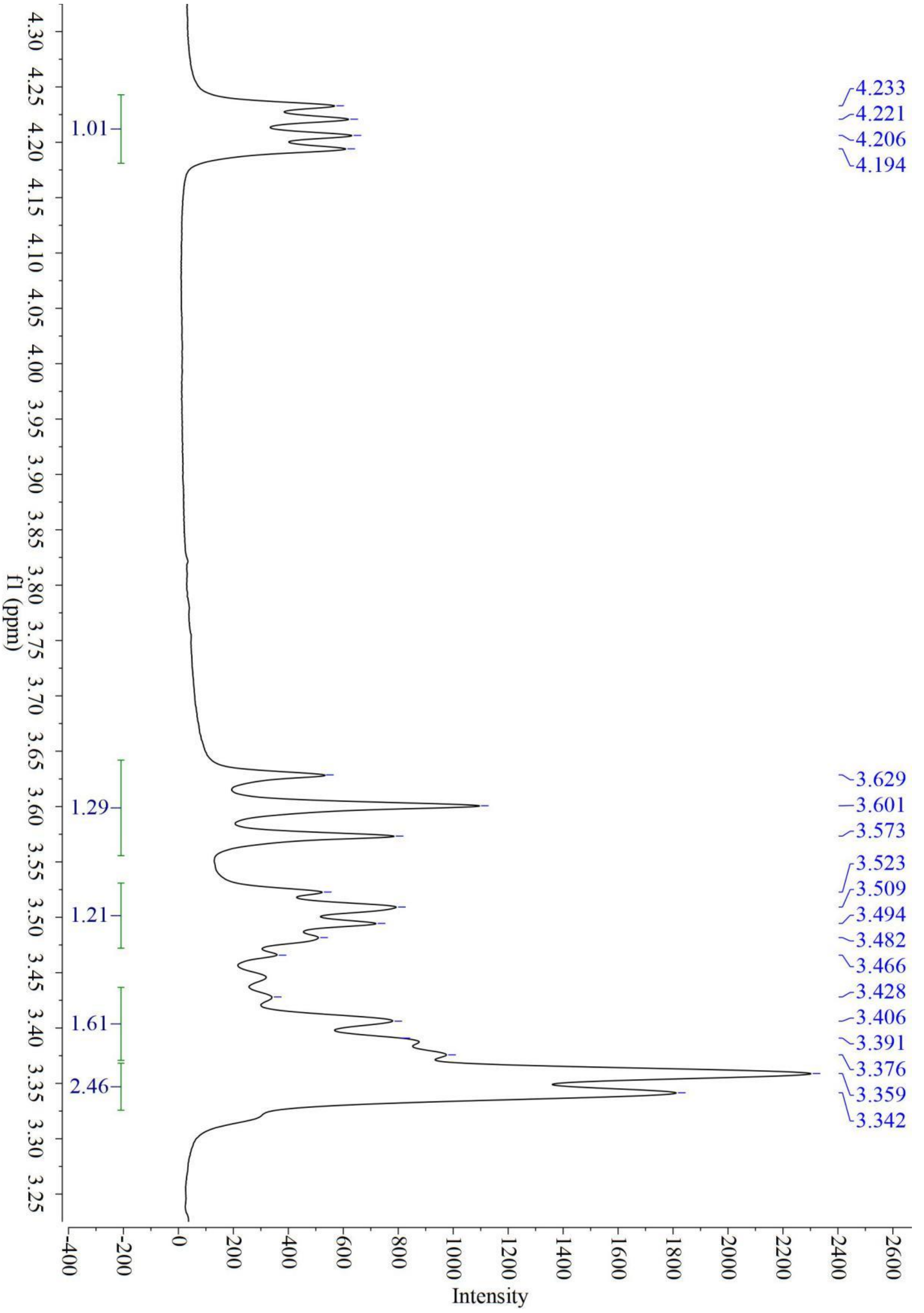

Figure S3. Magnified ^{1}H NMR ($CDCl_3$) spectrum of EL-19

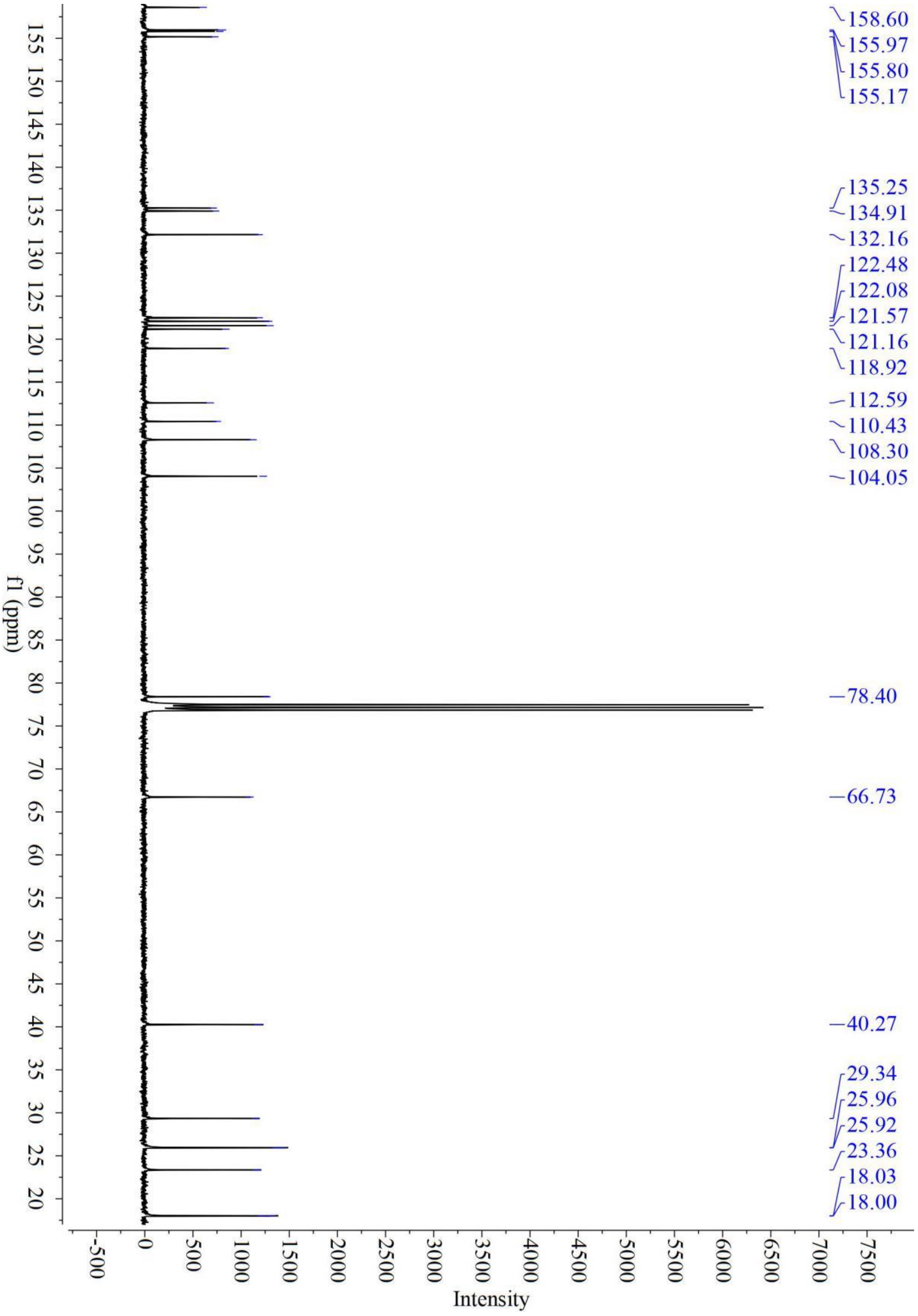

Figure S4. ^{13}C NMR ($CDCl_3$) spectrum of EL-19

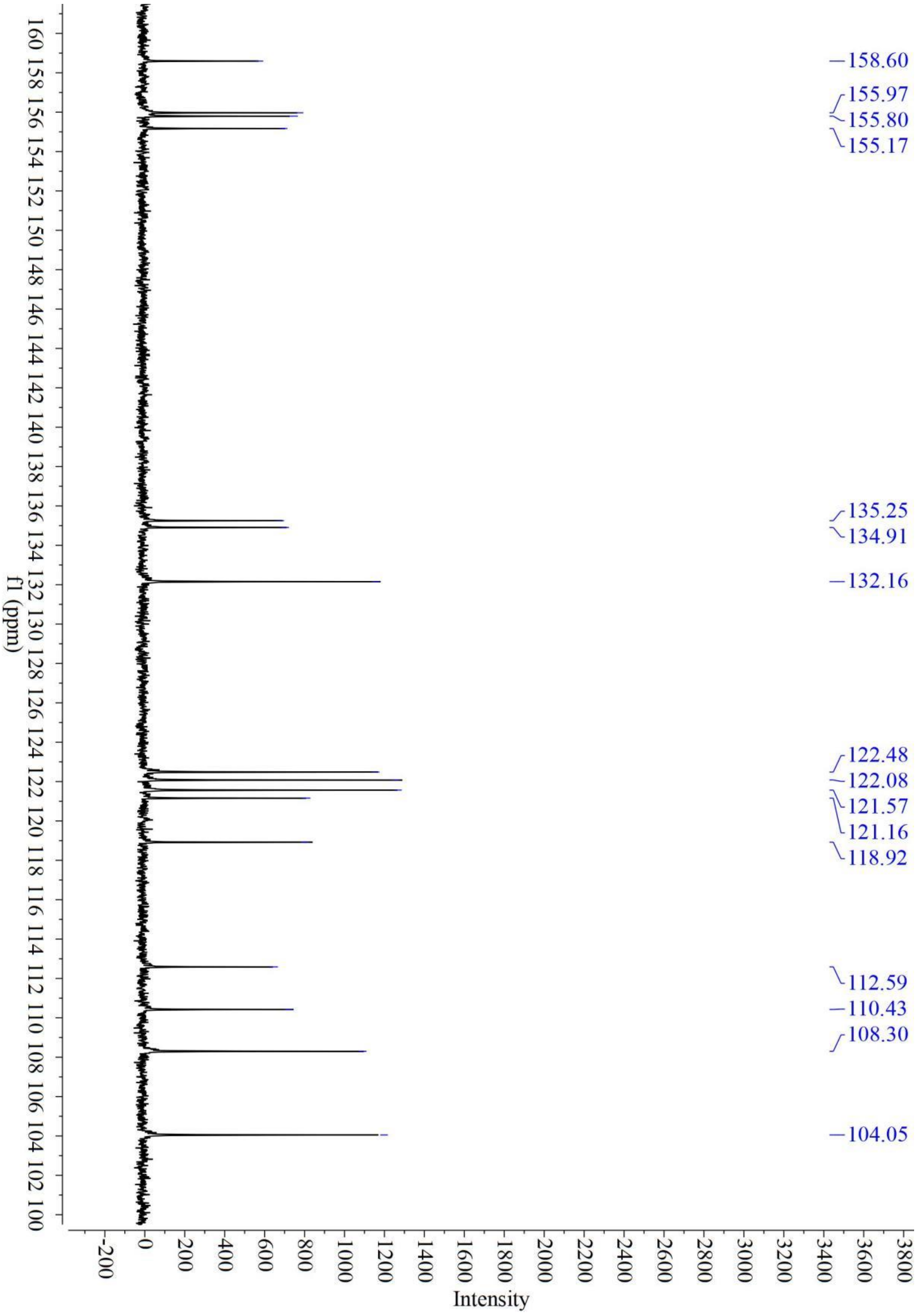

Figure S5. Magnified ^{13}C NMR ($CDCl_3$) spectrum of EL-19

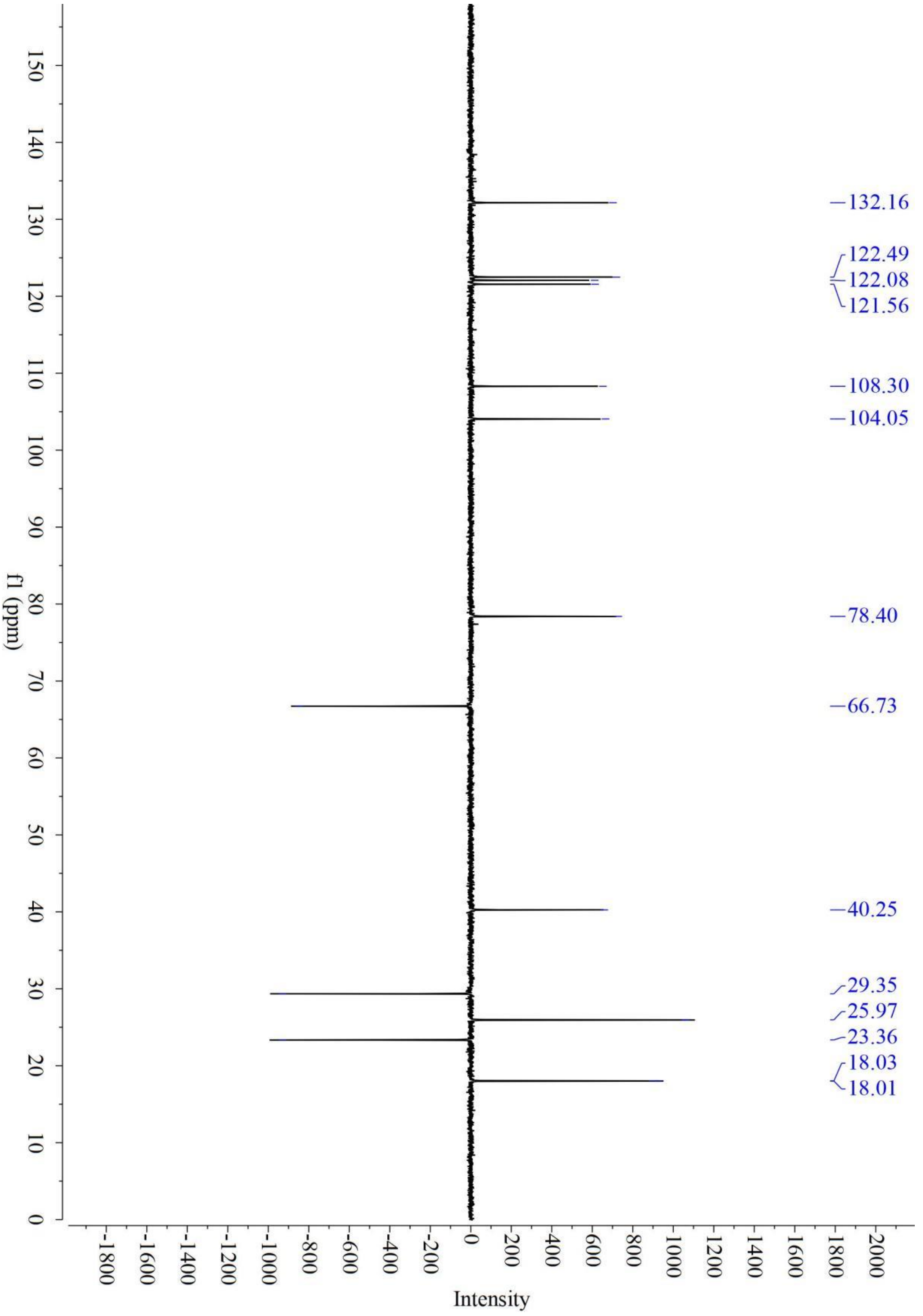

Figure S6. DEPT-135 ($CDCl_3$) spectrum of EL-19

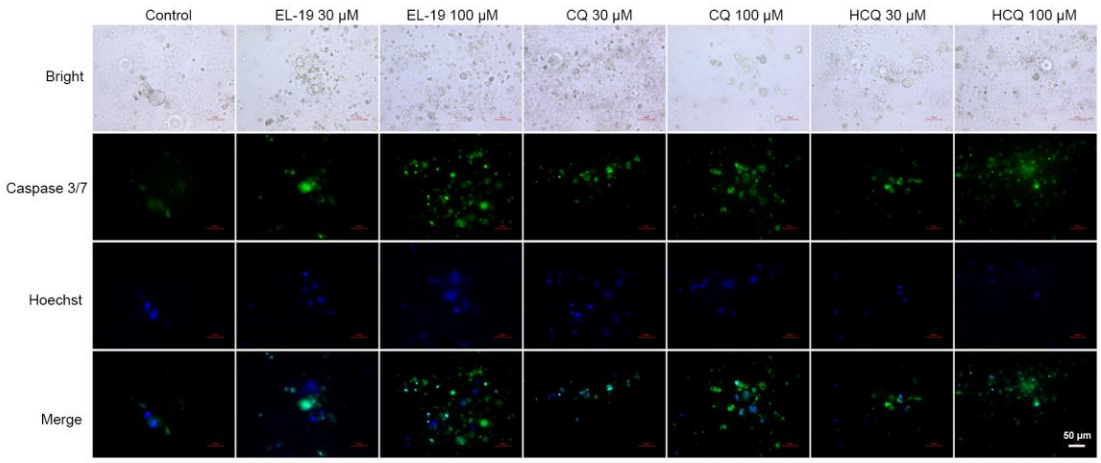

Figure S7. Apoptosis fluorescence staining on drug-treated OC organoids
